# Obesity as a Risk Factor for Complications and Mortality in Individuals with SARS-CoV-2: A Systematic Review

**DOI:** 10.3390/nu16040543

**Published:** 2024-02-16

**Authors:** Marielle Priscila de Paula Silva-Lalucci, Déborah Cristina de Souza Marques, Pablo Valdés-Badilla, Leonardo Vidal Andreato, Braulio Henrique Magnani Branco

**Affiliations:** 1 Interdisciplinary Laboratory of Intervention in Health Promotion, Cesumar Institute of Science, Technology, and Innovation, Maringá 87050-390, Paraná, Brazil; mariellepriscila@gmail.com (M.P.d.P.S.-L.); marques.deborah@hotmail.com (D.C.d.S.M.); 2 Graduate Program in Health Promotion, Cesumar University, Maringá 87050-390, Paraná, Brazil; 3 Department of Physical Activity Sciences, Faculty of Education Sciences, Universidad Católica del Maule, Talca 3530000, Chile; 4 Sports Coach Career, School of Education, Universidad Viña del Mar, Viña del Mar 2520000, Chile; 5 Higher School of Health Sciences, State University of Amazonas, Manaus 69065-001, Amazonas, Brazil; vidal.leo@hotmail.com

**Keywords:** COVID-19, overnutrition, hospitalization, oxygen inhalation therapy, clinical laboratory techniques

## Abstract

This systematic review aimed to analyze the available studies that identified overweight and/or obesity as a risk factor for mortality, use of respiratory support, and changes in biochemical markers in adults hospitalized with SARS-CoV-2. The PubMed, Web of Science, and Scopus databases were searched using PRISMA guidelines until January 2024. The protocol was registered with PROSPERO (code: CRD42024501551). Of the 473 articles, only 8 met the inclusion criteria (e.g., adult individuals aged 18 or over diagnosed with COVID-19 individuals with overweight and/or obesity). In addition, the Downs and Black tool was used to assess the quality of the studies. The studies analyzed totaled 9782 adults hospitalized for COVID-19, indicating that overweight and obesity are present in more than half of adults. Diseases such as diabetes mellitus and hypertension are more prevalent in adults with obesity. The systematic review also highlighted that a higher incidence of respiratory support is related to a higher incidence of hospitalization in intensive care units and that adults with overweight and obesity have a higher risk of mortality from COVID-19. Biochemical markers such as procalcitinin, C-reactive protein, and interleukin-6 are associated with the severity of COVID-19 infection. This systematic review exposed overweight and/or obesity as a risk factor for worse COVID-19 disease, as well as for the need for intensive care, respiratory support, mortality, and changes in essential blood markers.

## 1. Introduction

In recent years, the infection caused by the SARS-CoV-2 virus has caused one of the biggest public health problems in the world [1]. The pandemic spread rapidly and caused several health problems, resulting in excess morbidity and mortality, reaching 6,881,955 deaths [2,3]. COVID-19 is characterized by infection of the respiratory tract and flu-like symptoms are predominant; however, the infection can affect several areas (i.e., social, psychological, economic, and educational, among others), leading to cardiovascular (vasculitis, venous thrombosis, acute myocardial infarction, and myopericarditis) and pulmonary (pulmonary embolism, pulmonary fibrosis, pleurisy, and pneumonia) sequelae [4,5], as well as psychological sequelae, substantially affecting mental health [6].

The rapid and intense replication of SARS-CoV-2 deregulates the immune system, increasing the production of pro-inflammatory cytokines and contributing to the pathogenesis of COVID-19 [7]. In addition to high cytokine levels, laboratory tests related have identified hyperinflammation and tissue damage as predictors of disease severity [8]. Clinical studies have shown that altered levels of some blood markers may be related to the severity and mortality of individuals with COVID-19 [9].

Angiotensin-converting enzyme 2 (ACE2), the receptor used by SARS-CoV-2 to enter the host’s cells, is widely distributed throughout the human body, allowing the virus to target various organs, so the blood markers used to attest to the efficiency of these organs are altered [9,10]. The expression of ACE2 in pancreatic cells makes the pancreas a target for the virus, which can cause damage to the pancreatic islets, resulting in acute diabetes, and to the exocrine glands, causing acute pancreatitis, thus altering their blood markers [11]. Altered fasting glucose facilitates the hyperinflammation observed in the cytokine storm due to the deregulation of the innate immune system [11]. Elevated levels of inflammatory markers, such as C-reactive protein (CRP), have also been observed in individuals with COVID-19, as well as changes in renal markers due to the high expression of ACE2 in glomerular cells [12].

Although COVID-19 is a multisystem disease, obesity has been associated with the worst prognosis for this disease, supposedly as a result of inflammation, hormonal dysregulation, and indirectly through underlying diseases such as hypertension and diabetes [13,14]. Therefore, obesity has been reported as a significant risk factor for the SARS-CoV-2 virus [2]. As a result, these changes may predestine the management of COVID-19 disease [1]. The myriad concerns around the world have prompted public health providers to work to minimize the harmful effects and mitigate the impact of the increased morbidity and mortality associated with COVID-19 infection in people with obesity, as some studies show that hospitalization, mortality, and mechanical ventilation rates are increased in individuals with overweight [13,15]. In addition, class II obesity (≥35 kg/m^2^) is associated with a higher risk of ICU admission and the need for IMV [16]. The risk of mortality in individuals with obesity is higher compared to the non-obesity population [15].

Given the aspects listed, it is emphasized that the impacts of COVID-19 on people with obesity go beyond the increased risk for severe cases of the disease [17]. In this sense, people with obesity hospitalized for COVID-19 tend to lose a large proportion of lean mass due to physical inactivity and hospital nutritional therapy, as well as low-grade inflammation [18]. In addition, there is also a reduction in cardiorespiratory capacity in people with obesity hospitalized for COVID-19 [19,20,21]. Considering the above, this systematic review aimed to analyze the available studies that identified overweight and/or obesity as a risk factor for mortality, the use of respiratory support, and changes in biomarkers in adults hospitalized with SARS-CoV-2.

## 2. Materials and Methods

### 2.1. Protocol and Registration

The conduct of this systematic review followed the Preferred Reporting Items for Systematic Reviews and Meta-analyses (PRISMA) protocol [22], which corresponds to a checklist of 27 items designed to facilitate the development and reporting of a robust protocol for systematic reviews or meta-analyses. The protocol was registered in the International Prospective Register of Systematic Reviews (PROSPERO) under CRD42024501551.

### 2.2. Inclusion and Exclusion Criteria

The inclusion criteria for this systematic review were: (i) original articles written in English, Spanish, or Portuguese; (ii) that were published up to January 2024; (iii) that recruited adult individuals aged 18 or over of both sexes; (iv) that included individuals who were overweight and/or obese, according to the definitions presented by the studies based on BMI; (v) that included hospitalized individuals with a confirmed diagnosis of COVID-19 by real-time polymerase chain reaction (real-time PCR); (vi) that presented studies with an experimental design: cross-sectional, retrospective, and/or prospective; (vii) that the object of the study included individuals with overweight and/or obesity as a risk factor for COVID-19 complications; and (viii) studies with 60% or more of the total score when the Downs and Black scale was determined for more considerable reliability of the results [23,24]. On the other hand, the exclusion criteria were: (i) duplicate articles; (ii) case studies; (iii) Mendelian randomization analysis; (iv) studies linking COVID-19 with other underlying diseases (cancer, diabetes mellitus, hepatic steatosis, cardiovascular diseases, polycystic ovary, anorexia, influenza, human immunodeficiency virus, tuberculosis) or with pregnant women; (v) studies with associated interventions (e.g., psychological intervention, bariatric surgery), psychological intervention, bariatric surgery, vaccination, nutritional therapy/support); and (vi) studies with partial data from individuals without COVID-19.

### 2.3. Search Strategy

The search process was conducted in January 2024 using PubMed, Web of Science (core Collection), and Scopus. The Medical Subject Headings (MeSH) of the National Library of Medicine of the United States of America used unbiased language terms related to COVID-19, obesity, and biomarkers. The search string used was as follows: (“biomarkers” OR “inflammation” OR “lipid metabolism” OR “lipid metabolism disorders” OR “diabetes mellitus” OR “glycemic control” OR “C-reactive protein” OR “kidney function tests” OR “liver function tests” OR “electrolytes” OR “pancreatic function tests”) AND (“COVID-19” OR “SARS-CoV-2” OR “post-acute COVID-19 syndrome”) AND (“body composition” OR “body fat” “sarcopenia” OR “muscle strength” OR “muscles” OR “body mass index” OR “nutritional status” OR “anthropometry”) AND (“obesity” OR “obesity, abdominal” OR “overweight” OR “excess weight”). Citation alarms were set so that the principal researcher automatically received emails about the latest updates to the search terms used in the database (to include the most recent studies in the review). These updates were received daily (if available), and studies were eligible for inclusion until the start of manuscript preparation (11 January 2024). After the formal systematic searches, additional manual searches were carried out by consulting the reference lists of the included studies, and previous reviews and meta-analyses were reviewed to detect studies potentially eligible for inclusion.

### 2.4. Study Selection and Data Extraction

The studies were exported to EndNote reference manager software (version X9, Clarivate Analytics, Philadelphia, PA, USA). Three authors (MPPSL, DCSM, and BHMB) carried out the process independently. Disagreements between reviewers regarding the study conditions were resolved by a fourth author (PV-B). Subsequently, potentially eligible studies were reviewed in full text, and the reasons for excluding those not meeting the selection criteria were reported. Data (authors and year of publication, country of origin of the study, study design, study period, age group or the average age of the sample, gender, sample size, mortality rate, nutritional status, use of respiratory support, biomarkers, and main results obtained) from the studies were extracted by three authors (MPPSL, DCSM, and BHMB) independently, using a form created in Microsoft Excel (Version 2401, Microsoft Corporation, Redmond, WA, USA).

### 2.5. Methodological Quality Assessment

This phase aimed to detect the methodological quality in the selected studies through the Downs and Black [25] tool, which could eventually lead to the exclusion of the selected studies. Two authors (MPPSL and DCSM) carried out this process independently. A third author (BHMB) resolved possible disagreements between the reviewers. This reliable tool has been widely used in health research [25]. The instrument consists of 27 items, relating to reporting (10 items), external validity (3 items), internal validity bias (7 items), internal validity confounding (selection bias) (6 items), and statistical power (1 item), allowing a study to be rated between 0 and 32 points. The complete list is usually applied for randomized studies, whereas for non-randomized studies, it is reduced to 17 criteria after excluding items 9, 13, 14, 17, 19, 22, 23, 24, 26, and 27, which are not applicable in non-randomized studies, leading to a maximum score of 17 points [26]. In this way, the original non-randomized controlled trials and descriptive studies positively evaluated 60% (10 points or more out of 17) of the criteria selected and included in the subsequent analyses since they presented a moderate to high methodological quality [23,25,26].

### 2.6. Data Extraction

The following data were obtained and analyzed from the selected studies: (i) authors and year of publication, (ii) country of origin of the study, (iii) study design, (iv) study period, (v) age group or the average age of the sample, (vi) gender, (vii) sample size, (viii) mortality rate, (ix) nutritional status, (x) use of respiratory support, (xi) biomarkers, and (xii) main results obtained.

## 3. Results

### 3.1. Study Selection

**Figure 1:**
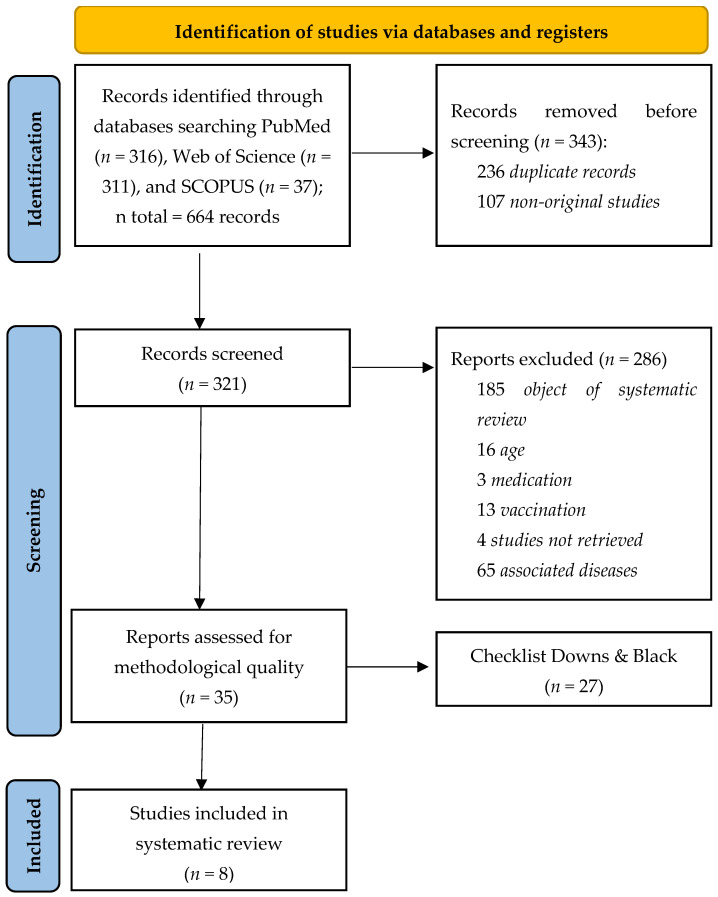
Flowchart of the study selection process.

The search process is shown in Figure 1. A total of 644 records were found in the study identification phase in PubMed (*n* = 316), Web of Science (*n* = 311), and Scopus (*n* = 37). In the screening phase, duplicate studies (*n* = 236) and non-original studies (*n* = 107) were excluded. A total of 321 full-text studies were analyzed, 185 of which were excluded because they did not meet the object of systematic review, 16 because they were studies with children under 18 years old, 3 because they were studies on medication (drugs), 13 because they were studies related to COVID-19 vaccination, 4 studies were not retrieved, and 65 studies were excluded because related COVID-19 to other underlying diseases. The remaining 35 studies were assessed for methodological quality using the Downs and Black scale. Of them, 27 studies were excluded due to low methodological quality, leaving 8 studies that met all the selection criteria [27,28,29,30,31,32,33,34].

### 3.2. Methodological Quality Assessment

Of the 35 studies selected for the methodological assessment using the Downs and Black scale [27,28,29,30,31,32,33,34,35,36,37,38,39,40,41,42,43,44,45,46,47,48,49,50,51,52,53,54,55,56,57,58,59,60,61], 1 study scored 6/17 [35], 3 scored 7/10 [36,37,38], 11 scored 8/17 [39,40,41,42,43,44,45,46,47,48,49], 12 scored 9/17 [50,51,52,53,54,55,56,57,58,59,60,61], 7 scored 10/17 [27,28,29,30,32,33,34], and 1 study scored 11/17 [31]. Only eight studies obtained 60% or more of the total scale score (17 points) [27,28,29,30,31,32,33,34]; therefore, they were selected to be analyzed in this systematic review (see Supplementary Table S1).

### 3.3. Study Characteristics

One study was carried out in Serbia [29], one in Turkey [30], one in Brazil [31], one in China [33], and four in the United States of America [27,28,32,34]. These studies analyzed nutritional status, use of oxygen support, mortality rate, and biochemical profiles in adults hospitalized with SARS-CoV-2.

Regarding the main comorbidities of COVID-19 positive adults, diabetes mellitus (DM) was also present in eight studies, with a prevalence ranging from 2% to 53% [27,28,29,30,31,32,33,34], chronic obstructive pulmonary disease was present in eight studies, ranging from 3.2% to 25.6% [27,28,29,30,31,32,33,34], systemic arterial hypertension (SAH) was present in seven studies, ranging from 31% to 83% [27,29,30,31,32,33,34], and cardiovascular diseases (acute myocardial infarction, congestive heart failure, chronic heart disease, coronary artery disease, and atrial fibrillation) were present in seven studies, ranging from 5.3% to 32.5% of the sample [27,28,30,31,32,33,34].

### 3.4. Sample Characteristics

The studies analyzed totaled 9782 adults hospitalized for COVID-19, with an average age of 63.3 years, 51.1% male participants, and a mortality rate of 17.9%. The characteristics of the studies and the sample are shown in Table 1.

### 3.5. Categorization by Nutritional Status and Use of Respiratory Support

Seven studies analyzed in this systematic review categorized BMI according to the cut-off points adopted by the World Health Organization (WHO) [27,28,29,30,31,32,34], in which adults are classified as: (i) underweight: <18.5 kg/m^2^, (ii) normal weight: 18.6–24.9 kg/m^2^, (iii) overweight: 25–29.9 kg/m^2^, (iv) obesity class I: 30–34.9 kg/m^2^, (v) obesity class II: 35–39.9 kg/m^2^, and (vi) obesity class III: >40 kg/m^2^ [62]. The study of Zeng et al. [33] is in line with the Chinese consensus on nutritional status, in which underweight is defined as BMI: ≤18.5 kg/m^2^, normal weight: 18.5–23.9 kg/m^2^, overweight: ≥24–27.9 kg/m^2^, and obesity is defined as ≥28 kg/m^2^ [63] (Table 2).

Of the eight studies selected, only two did not report the use of respiratory support [32,33]. The use of respiratory support reached 91.9% of adults hospitalized due to COVID-19 [29]. Stratification of the use of oxygen support occurred in five of the selected studies, with one article reporting the use of mechanical ventilation (MV) in 34% [27] and IMV in 11% of hospitalized adults [30]; the use of orotracheal intubation as oxygen support was reported in two studies, in 3.6% and 22% of adults, respectively [28,34]. Only one study stratified respiratory support into more than one type. Gil et al. [31] reported using oxygen therapy in 55.9% of the patients, non-invasive mechanical ventilation (NIMV) in 7.5% of patients, and IMV in 3.8% of patients; the data are presented in Table 2. Stevanovic et al. [29] found that 91.9% of hospitalized adults used respiratory support, of which 38.4% were overweight and 39.3% were obese. Nakeshbandi et al. [34] found that 74.1% of inpatients were on respiratory support, and 45.9% were obese.

### 3.6. Biochemical and Hematological Markers

Blood biochemical markers were present in six studies (Table 3), with C-reactive protein (CRP) showing a variation in the results measured in patients, with values ranging from 2.33 mg/L to 100 mg/dL; CRP was the most analyzed in the studies [28,30,31,32,33,34], followed by D-Dimer, which was present in five studies, with results ranging from 2383.8 ng/mL to 1.5 mg/dL [27,30,31,32,33] and lactate dehydrogenase (LDH), which was present in four studies, with results ranging from 179.10 U/L to 780 U/L [27,29,30,33]. Ferritin was analyzed in three studies [27,29,30], and creatinine was analyzed in three studies [27,31,33], with results ranging from 219.8 ng/mL to 877 ng/mL and 64.5 µmol/L to 1.4 mg/dL, respectively. Albumin [30,33], fasting blood glucose [30,33], urea [31,33], and procalcitonin (PCT) [29,30] were present in two studies each, with results of 31.26 g/L and 39 g/dL, 4.89 mmol/L and 127 mg/dL, 51.8 mg/dL and 4.41 mmol/L, and 0.089 µg/L and 0.142 µg/L, respectively. The biochemical markers analyzed in three studies were alanine aminotransferase (ALT) (23.3 U/L) [33], aspartate aminotransferase (AST) (19.8 U/L) [33], total bilirubin (9.50 U/L) [33], interleukin-6 (IL-6) (67.0 pg/mL) [29], gamma-glutamyl transferase (GGT) (30.2 U/L) [33], fibrinogen (6.59) [29], calcium (8.86 mmol/L) [29], direct bilirubin (3.30 U/L) [33], uric acid (287.43 µmol/L) [33], prothrombin time (PT) (12.83 s) [33], partially activated thromboplastin time (aPTT) (28.03 s) [33], and hemoglobin A1c (6.6%) [27].

Hematological markers were present in four studies (Table 3), with the absolute lymphocyte count [27,30,31,33], with values of 1.3 × 103/mm^3^, 1.2 × 10^9^/L, 26.28%, and 0.9 K/µL, respectively, the absolute neutrophil count [27,30,31,33], with values of 6.6 × 10^3^/µL, 3.78 × 10^9^/L, 63.3%, and 6.1 K/µL, respectively, hemoglobin [30,31,33], with values of 12.6 g/L, 123.93 g/L, and 13.2 g/dL, respectively, and absolute total leukocyte count in three studies [27,30,33], with results of 5.70 × 10^9^/L, 5.9 × 10^9^/L, and 7.7 K/µL respectively. Only four studies did not analyze blood parameters [28,29,32,34].

## 4. Discussion

This systematic review aimed to analyze the available studies that identified overweight and/or obesity as a risk factor for mortality, the use of respiratory support, and changes in biochemical markers in adults hospitalized with SARS-CoV-2. The following outcomes were observed in this systematic review: (i) male adults are more likely to be admitted to hospital; (ii) overweight and obesity were present in more than half of all adults of the analyzed studies; (iii) pre-existing diseases were more prevalent in adults with obesity; (iv) hypertension is the main comorbidity found among adults with COVID-19; (v) diabetes mellitus was the second most common comorbidity among adults; (vi) it was observed that a higher incidence of respiratory support use was related to a higher incidence of ICU admission; (vii) adults with overweight or obesity had an increased risk of mortality from COVID-19, and (viii) inflammatory markers such as PCT, CRP, and IL-6 are associated with the severity of COVID-19 infection.

Our systematic review was based on data from eight studies investigating COVID-19-positive adults [27,28,29,30,31,32,33,34]. All the selected studies showed that males are more likely to be admitted to hospital; males represented around 51.1% of all adults with COVID-19. Similar results were also presented by Huang et al. [64], who found that males are more likely to be infected than females. This response may occur because the sex hormone may play a role in the expression of the ACE2 receptor [65]. Older people and adults with pre-existing diseases are more susceptible to SARS-CoV-2, which may be associated with a higher frequency of comorbidities [66].

Chronic diseases, such as obesity, share several standard characteristics with infectious diseases, such as a pro-inflammatory state and attenuation of the innate response [67]. According to the WHO, obesity is a pandemic [68]. Estimates for the global levels of overweight (BMI ≥ 25 kg/m^2^) suggest that more than 4 billion people could be affected by 2035, reflecting an increase from 38% of the world’s population [69]. Obesity (BMI ≥ 30 kg/m^2^) alone will increase from 14% to 24% of the population over the same period, so one in four people will live with the disease [69]. In the Americas, 47% of all men and 49% of all women will be obese by 2035 [69]. In the European region, 35% of all women and 39% of men will be obese in the same period [69]. The projected annual growth of adults with obesity between 2020 and 2035 is 28%, which could cause a financial impact on the health sector of USD 19.2 billion in 2035 [69]. Overweight and obesity will likely cost the global economy more than USD 4 trillion in potential income by 2035, which is almost 3% of global gross domestic product (GDP) [69].

Obesity is associated with a poor prognosis for COVID-19; excess body fat reduces the response to antiviral agents through the deficient functions of T cells and macrophages [70]. The risk of health problems and death from COVID-19 is up to four times higher in adults with obesity [71]. Studies have shown that the prevalence of hospitalization of individuals with obesity and COVID-19 has increased. Rottoli et al. [72] reported that 36.5% of hospitalized adults were overweight, and 21.6% were obese. Petrilli et al. [73] found that overweight and obesity accounted for 34.3% and 32.8% of inpatients, respectively. Gao et al. [74] described a 12% increase in the risk of severe COVID-19 for each unit increase in BMI. In this systematic review, overweight and obesity were present in more than half of adults, with values ranging from 30% to 41% and 13.2% to 43%, respectively.

As previously described, obesity is a risk factor for the onset of other diseases (and these comorbidities are mainly responsible for increased mortality rates, decreased life expectancy, and quality of life) and, when associated with COVID-19, can lead to the development of severe COVID-19. Paravidino et al. [75] described that comorbidities for both adults aged <60 years and ≥60 years were more prevalent in adults with normal weight/overweight or obesity, with hypertension (55% and 77.9%) and diabetes mellitus (31.4% and 48.7%), respectively, standing out. Adults with multiple underlying medical conditions have a higher risk of severe COVID-19 disease [76]. In this systematic review, the comorbidities of adults with COVID-19 were analyzed, but only three studies analyzed the correlation between BMI and comorbidities. The studies reported that all pre-existing diseases were more prevalent in adults with obesity compared to the overall study population [30,33,34].

Systemic arterial hypertension (SAH) is currently the leading risk factor for morbidity and mortality worldwide and is a condition often observed in association with obesity [77,78]. Hypertension is the most frequent comorbidity in adults with COVID-19 and has been identified as a significant risk factor for the increased severity and mortality associated with COVID-19 [76]. Hypertension plays a vital role in regulating the renin–angiotensin–aldosterone system (RAAS) and immune responses, leading to the release of cytokines and increased inflammation [79]. Obesity aggravates hypertension by activating the RAAS, leading to the increased formation of angiotensin II, which induces vasoconstriction and aldosterone production, leading to salt and water retention [77]. A study analyzing 138 hospitals in China showed that the prevalence of hypertension was 31.2% among adults with COVID-19; in addition, 58.3% of hypertensive adults were admitted to the ICU, compared to 21.6% of normotensive adults [80]. A French study included 134,209 adults hospitalized with COVID-19, where 49.6% had hypertension and one in four had obesity (23.9%). IMV was recurrent in hospitalized adults with obesity [81]. This systematic review shows that hypertension is the main comorbidity found among adults with COVID-19, with values ranging from 62% to 67.6%; however, none of these studies correlated the prevalence of hypertensive adults with BMI [27,29,31,32]. In contrast to previous studies, Agca et al. [30], Zeng et al. [33], and Nakeshbandi et al. [34] correlated hypertension with BMI, with values ranging from 27% to 85% for overweight people and 44% to 87% for people with obesity.

In addition to SAH, studies have reported a higher risk of severe COVID-19 in diabetic adults due to the impairment of the immune system [82,83]. Diabetes mellitus (DM) is a chronic syndrome of multiple etiology resulting from the lack and/or inability of insulin to exert its effects adequately [84]. Diabetic adults have increased ACE2 expression, leading to a higher risk of infection by the COVID-19 virus since ACE2 is the receptor for SARS-CoV-2 [85]. ACE2 is expressed in pancreatic beta cells, the cells responsible for hormones such as insulin, which controls blood glucose [86]. In addition, furin protease, which mediates furin endoprotease and cleaves the virus’s S protein, is expressed at high levels in diabetic patients [84,87].

The results showed that DM was the second most common comorbidity found in this systematic review, with the incidence of DM in adults with obesity ranging from 20.6% to 57% [27,30,33,34]. A previous meta-analysis of 33 studies with 16,003 adults showed that diabetic adults with COVID-19 are twice as likely to develop severe COVID-19 and die compared to non-diabetics. They are thus more likely to develop acute respiratory distress syndrome (ARDS), need IMV, and be admitted to the ICU [88]. Another meta-analysis of 40 studies involving 18,012 adults with COVID-19 showed that DM leads to a 2.3-fold increase in symptom severity and a 2.5-fold increase in mortality associated with COVID-19 [89].

The increase in respiratory complications resulting from COVID-19, the need for ICU admission, and the use of mechanical ventilation appear to be associated with increased BMI [90]. Physiologically, increased body weight increases the mechanical pressure on the chest and abdomen, thus compromising lung function [91]. Thus, oxygen consumption decreases and, consequently, causes a decrease in expiratory reserve volume, functional capacity, and lung compliance [92,93]. The abnormal release of cytokines in obesity can impair the immune response and influence the lung parenchyma and bronchi [94].

A retrospective cohort study of 200 COVID-19-positive adults seen in a United States emergency room found that adults with severe obesity were more likely to undergo intubation as BMI progressed, with 16.4% of adults with a BMI ≥ 25 kg/m^2^ and 34.8% of adults with a BMI ≥ 35 kg/m^2^ undergoing intubation [95]. Simonnet et al. [96] confirmed the results found previously; the proportion of adults who required IMV increased with BMI categories and was higher in adults with BMI ≥ 35 kg/m^2^, reaching almost 90% of adults. A systematic review supporting the above information found that the rate of oxygen support use ranged from 3.6 to 91.9%, where the higher incidence of oxygen support use was related to a higher incidence of ICU stay [29]. Nakeshbandi et al. [34] observed that the highest prevalence of intubation was related to overweight and adults with obesity, with incidences of 24% and 28%, respectively.

Obesity is known to contribute to an increased risk of severity and mortality from COVID-19 infection [15]. Obesity alters the mechanical properties of the lungs and chest wall, thus promoting fibrosis, contraction, and vasoconstriction [97,98]. Obesity can also increase the inflammatory process caused by COVID-19 by up-regulating cytokines, contributing to worse outcomes in adults with COVID-19 [99,100]. In addition, increased production of these cytokines is associated with alveolar damage [101]. Obesity is associated with hypercoagulation and an increased risk of arterial thrombosis and venous thromboembolism [102,103]. Finally, increased expression of ACE2 receptors in adipose tissue increases susceptibility to SARS-CoV-2 infection, the risk of severe disease, and mortality in obese adults [104,105]. In our systematic review, overweight adults or adults with obesity had an increased risk of mortality from COVID-19, with values ranging from 4% to 40% in adults with obesity; however, a higher rate occurred in overweight adults, reaching 54% of deaths [30,33,34]. In a study covering 142 countries, a robust positive association was observed between the percentage of obese adults and COVID-19 mortality [106]. In another cohort study involving 200 adults, it was observed that, during hospitalization, 24% died, with higher rates among adults with severe obesity (34.8%) [95]. In another American study, similar results were also observed, with severe obesity being associated with higher mortality from COVID-19 [107].

Finally, several biomarkers are currently being used to predict the severity of COVID-19. Inflammatory markers such as PCT, CRP, and IL-6 are reported to be associated with the severity of COVID-19 infection [108]. Markers such as ferritin, D-dimer, LDH, liver enzymes (ALT, AST, GGT, alkaline phosphatase (ALP), and total bilirubin), and kidney functions (creatinine, albumin, and total serum protein) are also monitored in patients suffering from COVID-19 [109]. Increased pro-inflammatory cytokines, such as IL-6 and TNF-α, have been observed in adults with severe COVID-19 and are significantly associated with mortality [110]. PCT is an inflammatory marker of the critical phase of COVID-19 infection; high PCT values have been associated with an approximately fivefold increased risk of severe COVID-19 [109]. The many pathological processes in COVID-19 include hyperinflammation, cytokine storms, and deregulation of the coagulation pathway, among others. Therefore, laboratory tests can help assess the prognosis of the disease, determine the appropriate therapeutic options, and scrutinize the response to treatment.

Adults with COVID-19 are likely to need rehabilitation intervention during and directly after hospitalization [111]. Rehabilitation is a multidisciplinary intervention that aims to improve functional capacity, increase the quality of life, facilitate social reintegration after hospitalization, reduce persistent symptoms, and improve the ability to perform the activities of daily living [112]. Sordi et al. [113] evaluated the effect of a multi-professional intervention (nutritional, psychoeducational, and physical exercise intervention) on body composition, physical fitness, and biochemical markers in overweight COVID-19 survivors (BMI ≥ 25 kg/m^2^) with different symptoms. After the interventions, the authors reported that the moderate COVID-19 group showed improvement in the dynamic muscle strength of the lower and upper limbs, maximum lumbar isometry—traction force, flexibility, and markers such as albumin, CRP, fasting glycemia, and triglycerides; for the severe COVID-19 group, improvements were seen in the dynamic muscle strength of the lower limbs and lower CRP and triglyceride values; for the control group, improvements were seen in abdominal repetitions, CRP, fasting glucose, TC, and triglycerides.

In another study, Perli et al. [21] assessed the body composition, cardiorespiratory fitness, and long-term symptoms of overweight adults affected by COVID-19. The most prevalent long-term symptoms were memory deficit (66.7%), lack of concentration (51.7%), fatigue (65.6%), and dyspnea (40%). The Bruce test showed a time effect with increased distance covered after 1 year only for the severe/critical group. Percutaneous oxygen saturation (SpO_2_) was significantly lower in the severe/critical group up to 5 min after the Bruce test when compared to the mild group, and diastolic blood pressure at the end of the Bruce test was significantly higher in the severe/critical group when compared to the mild group. A time effect was observed for body composition, with increased lean mass, skeletal muscle mass, fat-free mass, and lean mass only for the severe/critical group. Because of this, multi-professional interventions could be an efficient tool for reversing the inflammatory process and promoting improvements in daily living activities and quality of life [21].

Among the limitations of this systematic review are: (i) the populations studied differed in their comorbidities and severity, (ii) body weight and height were self-reported or reported by family members at the time of hospital admission, (iii) the definition of BMI categories is not consensual, the WHO defines overweight as BMI ≥ 25 kg/m^2^ and obesity as BMI ≥ 30 kg/m^2^, but the cut-off values for the Chinese population define overweight as BMI 24.0–27.9 kg/m^2^ and obesity as BMI ≥ 28 kg/m^2^, (iv) some covariates had missing data, which could have influenced the results, and (v) only three studies presented their data according to BMI. In terms of strengths, we found (i) methodological quality above 60% in the studies analyzed, (ii) methodological processes that followed the PRISMA, PROSPERO, and Downs and Black tools, and (iii) the use of three databases: PubMed, Scopus, and Web of Science (core collection). 

Consequently, these results may help draw up public health policies, especially in countries with a higher prevalence of individuals with obesity, as well as directing the actions of health professionals towards care aimed at integrality and humanization and promoting educational actions capable of making the population aware of the importance of self-care and healthy lifestyles.

## 5. Conclusions

This systematic review exposed overweight and/or obesity as a risk factor for worse COVID-19 disease, as well as for the need for intensive care, respiratory support, mortality, and changes in significant blood markers. It should be noted that obesity is a chronic non-communicable disease present in all age groups that can be prevented and that healthy lifestyles can reduce the severity of COVID-19 infection.

Thus, it is understood that health professionals should pay special attention to overweight and obese individuals. Adopting a healthy lifestyle, including adequate and healthy eating and physical activity, is essential for maintaining and recovering a healthy weight. Individuals hospitalized with obesity must be carefully monitored and managed quickly and effectively so that therapeutic interventions by a multidisciplinary team can be carried out in the best way, promoting a reduction in morbidity and mortality in these individuals.

## 6. Practical Applications

The etiology of obesity is complex and multifactorial, resulting from the interaction of genes, environment, lifestyle, and emotional factors. It is, therefore, essential to assess the causes that lead people to gain body weight, to investigate the possible associated morbidities, and, above all, to discuss actions that seek to promote the health of people with overweight and obesity. All interventions that treat obesity in all its forms must be multidisciplinary. Food and nutritional guidance for people who are overweight and/or obese, aimed at reducing the consumption of foods rich in sugar and fat, combined with regular physical activity, are essential strategies in the treatment of obesity. Given this, governmental and non-governmental strategies in the early phases of life are recommended to reduce the impact caused by chronic diseases, especially obesity. In addition to this, micro, meso, and macro strategies are recommended, with the creation of specialized treatment centers with an approach regarding health in general, how to improve health, and how to promote health promotion in primary and secondary education, as well as in the college and university. Many diseases could be avoided by education. Thus, the educational approach should be considered in the life cycle, i.e., children, adolescents, young adults, middle-aged adults, and older adults. Finally, our efforts may also be directed at pregnant women and children (more extended actions), focusing on health education to change unhealthy habits.

## Figures and Tables

**Table 1:** Characteristics of the studies and the sample.

Study	Country	Study Design	Study Period	Age Group or Mean (Years)	Sex (%)	Adults	Mortality (%)
Zahid et al. [27]	United States	Retrospective study	03/2020–06/2020	64	F 39.0 M 61.0	1.274	NR
Salvy et al. [28]	United States	Retrospective study	03/2020–01/2021	<65 and ≥ 65	F 48.5 M 51.5	3.861	8.6
Stevanovic et al. [29]	Serbia	Prospective observational study	10/2021–12/2021	67	F 37.0 M 63.0	216	16.7
Agca et al. [30]	Turkey	Observational, cross-sectional, and retrospective study	03/2020–05/2020	54	F 42.6 M 57.4	284	8.0
Gil et al. [31]	Brazil	Prospective observational study	03/2020–10/2020	59.0 ± 15.0	F 50.0 M 50.0	186	6.5
Naaraayan et al. [32]	United States	Retrospective study	03/2020–05/2020	71	F 43.4 M 56.6	348	40.5
Zeng et al. [33]	China	Retrospective cohort study	02/2020–04/2020	58.5 ± 14.3	F 49.3 M 50.7	3.019	2.2
Nakeshbandi et al. [34]	United States	Retrospective cohort study	03/2020–04/2020	68.0 ± 15.0	F 48.0 M 52.0	504	43.0

Note: F = female; M = male; NR = not reported.

**Table 2:** Characteristics of nutritional status and use of oxygen support in the studies.

Studies	BMI kg/m² (Mean)	Categorization by BMI kg/m²	Respiratory Support (%) ^a^
Zahid et al. [27]	28.7	Overweight, 4.9% Obesity, 42.2%	34.0
Salvy et al. [28]	<65 years: 25.8≥65 years: 26.0	Overweight, 30.7% Obesity, 35.6%	3.6
Stevanovic et al. [29]	NR	Overweight, 38.4% Obesity, 39.3%	91.9
Agca et al. [30]	25.9	Overweight, 41.0% Obesity, 24.0%	11.0
Gil et al. [31]	29.5 ± 6.9	Obesity, 40.9%	Oxygen therapy, 55.9 NIMV, 7.5 IMV, 3.8
Naaraayan et al. [32]	NR	Overweight, 35.3% Obesity, 34.8%	NR
Zeng et al. [33]	NR	Overweight, 38.6% Obesity, 13.2%	NR
Nakeshbandi et al. [34]	NR	Overweight, 30.0% Obesity, 43.0%	22.0

Note: BMI = body mass index, NIMV = non-invasive mechanical ventilation, IMV = invasive mechanical ventilation, NR = not reported; ^a^ = respiratory support results represent the whole group, without division by BMI.

**Table 3:** Characteristics of the biochemical markers and hematological markers in the studies.

Studies	Laboratory Tests
Zahid et al. [27]	Biochemical markers: - CRP: 112 mg/L - creatinine: 1.2 mg/dL - LDH: 484 µ/L - D-dimer: 524 ng/mL - ferritin: 756.05 ng/mL - hemoglobin A1c: 6.6% Hematological markers: - leukocytes: 7.7 K/µL - lymphocytes: 0.9 K/µL - neutrophils: 6.1 K/µl
Salvy et al. [28]	NR
Stevanovic et al. [29]	Biochemical markers = CRP: 100.0 mg/dL - IL-6: 67.0 pg/mL - ferritin: 877.0 ng/mL - LDH: 780.0 U/L - fibrinogen: 6.59 - PCT: 0.142 µg/L
Agca et al. [30]	Biochemical markers = glycemia: 127.0 mg/dL - albumin: 39.0 g/dL - calcium: 8.86 mmol/L - LDH: 267.0 U/L - D-dimer: 0.61 µd/L - CRP: 47.7 mg/mL - PCT: 0.089 µg/L - ferritin: 219.8 ng/mL Hematological markers = leukocytes: 5.9 10⁹/L - Hb: 13.2 g/dL - platelets: 214 10⁹/L - lymphocytes: 1.2 10⁹/L - neutrophils: 3.78 10⁹/L
Gil et al. [31]	Biochemical markers = CRP: 92.3 mg/dL - urea: 51.8 mg/dL - D-dimer: 2383.8 ng/mL - creatinine: 1.4 mg/dL Hematological markers = Hb: 12.6 g/L - neutrophils: 6.6 10³/mm³ - lymphocytes: 1.3 10³/mm³ - platelets: 255.8 10³/mm³
Naaraayan et al. [32]	Biochemical markers = CRP: 176.4 mg/L - D-dimer: 1.5 mg/dL
Zeng et al. [33]	Biochemical markers = CRP: 2.33 mg/L - uric acid: 287.43 µmol/L - creatinine: 64.5 µmol/L - glycemia: 4.89 mmol/L - urea: 4.41 mmol/L - albumin: 37.26 g/L - ALT: 23.30 U/L - AST: 19.80 U/L - GGT: 30.20 U/L - LDH: 179.10 U/L - TBIL: 9.50 U/L - DBIL: 3.30 U/L - TP: 12.83 seg. - TTPa: 28.03 seg. - D-dimer: 0.42 mg/L Hematological markers = leukocytes: 5.70 10⁹/L - Hb: 123.93 g/L - platelets: 234.01 10⁹/L - lymphocytes: 26.28% - neutrophils: 63.3%
Nakeshbandi et al. [34]	NR

Note: PCT = procalcitonin, LDH = lactate dehydrogenase, CRP = reactive protein C, IL-6 = interleukin-6, Hb = hemoglobin, ALT = alanine aminotransferase; AST = aspartate aminotransferase; GGT = gamma-glutamyl transferase; TBIL = total bilirubin, DBIL = direct bilirubin, TP: prothrombin time, TTPa = partially activated thromboplastin time, NR = not reported.

## Supplementary Materials

The following supporting information can be downloaded at: https://www.mdpi.com/article/10.3390/nu16040543/s1, Table S1: Methodological quality assessment.
