# Supplementary material for: Obesity as a Risk Factor for Complications and Mortality in Individuals with SARS-CoV-2: A Systematic Review
Source: Nutrients. 2024 Feb 16;16(4):543. doi: 10.3390/nu16040543 (PMC10892776; doi:10.3390/nu16040543)
Supplement: Supplementary file 1 [file nutrients-16-00543-s001.zip › nutrients-2861485-supplementary.pdf]

Supplementary Table S1. Methodological quality assessment *.

Criteria	1	2	3	4	5	6	7	8	9	10	11	12	13	14	15	16	17	Total
**AlBahrani et al. [36]**	0	1	0	N/D	1	1	0	N/D	1	1	1	N/D	0	1	1	N/D	0	7
**Torrego-Ellacuría et al. [37]**	1	1	0	N/D	1	0	0	N/D	1	1	0	N/D	0	1	1	N/D	0	7
**Tong [38]**	1	1	0	N/D	1	0	1	N/D	0	1	0	N/D	0	1	1	N/D	0	7
**Tonietto et al. [50]**	1	1	1	N/D	1	0	1	N/D	1	1	0	N/D	0	1	1	N/D	0	9
**Yamamoto et al. [39]**	1	1	0	N/D	1	0	1	N/D	1	1	0	N/D	0	1	1	N/D	0	8
**Zahid et al. [27]**	1	1	1	N/D	1	1	1	N/D	1	1	0	N/D	0	1	1	N/D	0	**10**
**Caccialanza et al. [40]**	0	1	1	N/D	1	1	0	N/D	1	1	0	N/D	0	1	1	N/D	0	8
**Palaiodimos et al. [51]**	1	1	1	N/D	1	1	1	N/D	0	1	0	N/D	0	1	1	N/D	0	9
**Salvy et al. [28]**	1	1	1	N/D	1	1	1	N/D	1	1	0	N/D	0	1	1	N/D	0	**10**
**Stevanovic et al. [29]**	1	1	1	N/D	1	1	1	N/D	1	1	0	N/D	0	1	1	N/D	0	**10**
**Agca et al. [30]**	1	1	1	N/D	1	1	1	N/D	1	1	0	N/D	0	1	1	N/D	0	**10**
**Al-Salameh et al. [52]**	1	1	1	N/D	1	1	0	N/D	1	1	0	N/D	0	1	1	N/D	0	9
**Azarkar et al. [41]**	0	1	1	N/D	1	1	0	N/D	1	1	0	N/D	0	1	1	N/D	0	8
**Cai et al. [53]**	1	1	0	N/D	1	1	1	N/D	1	1	0	N/D	0	1	1	N/D	0	9
**Gao et al. [35]**	0	0	0	N/D	0	1	1	N/D	1	1	0	N/D	0	1	1	N/D	0	6
**Gil et al. [31]**	1	1	1	N/D	1	1	1	N/D	1	1	1	N/D	0	1	1	N/D	0	**11**
**Jayanama et al. [54]**	1	1	0	N/D	1	1	1	N/D	1	1	0	N/D	0	1	1	N/D	0	9
**Kang et al.[42]**	1	1	0	N/D	0	1	1	N/D	1	1	0	N/D	0	1	1	N/D	0	8
**Le Guen et al. [55]**	1	1	0	N/D	1	1	1	N/D	1	1	0	N/D	0	1	1	N/D	0	9
**Lucar et al. [56]**	1	1	0	N/D	1	1	1	N/D	1	1	0	N/D	0	1	1	N/D	0	9
**Martínez Urbistondo et al. [43]**	1	1	0	N/D	0	1	1	N/D	1	1	0	N/D	0	1	1	N/D	0	8
**Martinuzzi et al. [44]**	1	0	1	N/D	0	1	1	N/D	1	1	0	N/D	0	1	1	N/D	0	8
**McNeill et al. [45]**	1	1	1	N/D	0	0	1	N/D	1	1	0	N/D	0	1	1	N/D	0	8
**Naaraayan et al. [32]**	1	1	1	N/D	1	1	1	N/D	1	1	0	N/D	0	1	1	N/D	0	**10**
**Ninomiya et al. [57]**	1	1	1	N/D	0	1	1	N/D	1	1	0	N/D	0	1	1	N/D	0	9
**Okauchi et al. [58]**	0	1	1	N/D	1	1	1	N/D	1	1	0	N/D	0	1	1	N/D	0	9
**Pérez-Cruz et al. [46]**	1	1	0	N/D	1	1	0	N/D	1	1	0	N/D	0	1	1	N/D	0	8
**Ye et al. [59]**	0	1	1	N/D	1	1	1	N/D	1	1	0	N/D	0	1	1	N/D	0	9
**Zeng et al. [33]**	1	1	1	N/D	1	1	1	N/D	1	1	0	N/D	0	1	1	N/D	0	**10**
**Al-Sabah et al. [47]**	0	1	0	N/D	1	1	1	N/D	1	1	0	N/D	0	1	1	N/D	0	8
**Anderson et al. [48]**	1	1	0	N/D	1	1	0	N/D	1	1	0	N/D	0	1	1	N/D	0	8
**Kaeuffer et al. [60]**	1	1	0	N/D	1	1	1	N/D	1	1	0	N/D	0	1	1	N/D	0	9
**Mostaghim et al. [61]**	1	1	0	N/D	1	1	1	N/D	1	1	0	N/D	0	1	1	N/D	0	9
**Nakeshbandi et al. [34]**	1	1	1	N/D	1	1	1	N/D	1	1	0	N/D	0	1	1	N/D	0	**10**
**Rao et al. [49]**	1	0	1	N/D	0	1	1	N/D	1	1	0	N/D	0	1	1	N/D	0	8

Note: * According to the Downs & Black checklist. N/D: not determined; 1: complies; 0: does not comply. Criteria: 1: Is the hypothesis/objective of the study clearly described? 2: Are the main outcomes to be measured clearly described in the introduction or methods section? 3: Are the patients' characteristics in the study clearly described? 4: Are the interventions of interest clearly described? 5: Are the distributions of the main confounding factors in each group of subjects to be compared clearly described? 6: Are the main findings of the study clearly described? 7: Does the study provide estimates of random variability in the data for the main outcomes? 8: Have all important adverse events that may result from the intervention been reported? 9: Have actual probability values (e.g., 0.035 instead of <0.05) been reported for the main outcomes, except when the probability value is less than 0.001? 10: Are the sample selection procedures clearly described? 11: Were the study participants representative of the population from which they were recruited? 12: Was there an attempt to blind those who measured the results of the intervention? 13: Was it clear whether any of the study results were based on data mining (i.e., misuse of data analysis to present them as statistically significant)? 14: Were appropriate statistical tests used to assess the main outcomes? 15: Were accurate (valid and reliable) main outcome measures used? 16: Were patients recruited from the same population in different intervention groups (trials and cohort studies) or cases and controls (case-control studies)? 17: Was there an adequate adjustment for confounding in the analyses from which the main findings were drawn?
